# Wearable defibrillator use in heart failure (WIF): results of a prospective registry

**DOI:** 10.1186/1471-2261-12-123

**Published:** 2012-12-12

**Authors:** Andrew C Kao, Steven W Krause, Rajiv Handa, Darshak Karia, Guillermo Reyes, Nicole R Bianco, Steven J Szymkiewicz

**Affiliations:** 1 Mid America Heart and Vascular Institute, St. Luke’s Hospital, Kansas City, MO, USA; 2 Blessing Hospital, Quincy, IL, USA; 3 Metro Heart Group of St. Louis, St. Louis, USA; 4 Albert Einstein Medical Center, Philadelphia, USA; 5 Cardiology of San Antonio, PA, San Antonio, USA; 6 ZOLL Cardiac Management Solutions, Pittsburgh, PA, USA

**Keywords:** Heart failure, Wearable cardioverter defibrillator, Sudden cardiac death

## Abstract

**Background:**

Heart failure (HF) patients have a high risk of death, and implantable cardioverter defibrillators (ICDs) are effective in preventing sudden cardiac death (SCD). However, a certain percentage of patients may not be immediate candidates for ICDs, particularly those having a short duration of risk or an uncertain amount of risk. This includes the newly diagnosed patients, as well as those on the cardiac transplant list or NYHA class IV heart failure patients who do not already have an ICD. In these patients, a wearable cardioverter defibrillator (WCD) may be used until long term risk of SCD is defined. The purpose of this study was to determine the incidence of SCD in this population, and the efficacy of early defibrillation by a WCD.

**Methods:**

Ten enrolling centers identified 89 eligible HF patients who were either listed for cardiac transplantation, diagnosed with dilated cardiomyopathy, or receiving inotropic medications. Data collected included medical history, device records, and outcomes (including 90 day mortality).

**Results:**

Out of 89 patients, final data on 82 patients has been collected. Patients wore the device for 75±58 days. Mean age was 56.8±13.2, and 72% were male. Most patients (98.8%) were diagnosed with dilated cardiomyopathy with a low ejection fraction (<40%) and twelve were listed for cardiac transplantation. Four patients were on inotropes. There were no sudden cardiac arrests or deaths during the study. Interestingly, 41.5% of patients were much improved after WCD use, while 34.1% went on to receive an ICD.

**Conclusions:**

In conclusion, the WCD monitored HF patients until further assessment of risk. The leading reasons for end of WCD use were improvement in left ventricular ejection fraction (LVEF) or ICD implantation if there was no significant improvement in LVEF.

## Background

Sudden cardiac death (SCD) is believed to account for 40 to 70% of the mortality associated with congestive heart failure (HF), with progressive pump failure causing the majority of the remaining fatalities [1,2]. Current figures estimate that 20% of HF patients will die within one year of diagnosis [3], which represents an improvement over prior decades [4]. Thus, the sudden death rate in the first year of diagnosis is about 10%, mostly resulting from terminal ventricular arrhythmias. Further complicating the situation, some medications used in the treatment of HF (ie, inotropes and diuretics) can also contribute to ventricular tachycardia (VT) in patients [5]. Implantable cardioverter-defibrillators (ICDs) are effective in preventing SCD in HF due to ischemic and non-ischemic cardiomyopathy when cardiac function, as measured by ejection fraction (EF), is ≤ 35% [6-9]. HF patients on heart transplant lists have also been shown to benefit from ICDs [10-12]. However, ICDs may not be the most cost-effective [13], or the best method of reducing the incidence of sudden death in all HF patients, particularly in patients having a short (or uncertain) duration of risk or those with drug refractory NYHA class IV HF [14]. According to AHA/ACC/HRS guidelines for ICDs, newly diagnosed nonischemic dilated cardiomyopathy (NIDCM) patients should be medically optimized, a process that may take months, before implantation [15]. Conversely, wearable cardioverter defibrillators (WCD) such as the LifeVest® device (manufacturer: ZOLL, Pittsburgh, PA) can be used immediately to protect against SCD until long-term risk of sudden death is established or cardiac function improves.

The WCD (LifeVest®, ZOLL, Pittsburgh, PA) was FDA-approved in 2001 as an outpatient device after a clinical trial involving 285 out-of-hospital patients [16]. WCDs are intended for use in adult patients that have an increased risk of SCD if an ICD is not warranted. Since approval, more than 60,000 patients have used the LifeVest® on an outpatient basis for a wide variety of conditions. Like ICDs, WCDs are designed to detect and treat ventricular tachyarrhythmias without the need for bystander intervention. Unlike ICDs, they are completely external to the body and do not require surgical intervention. Thus, they are ideal for shorter-term applications where the risk of SCA is changing or uncertain. WCD use in the HF population is limited [16,17]. In order to gain further clinical experience with WCD use in this population, ZOLL sponsored the WIF (Wearable defibrillator use In heart Failure) registry. The primary purpose of the WIF registry was to determine the incidence of SCA in this population, the cause of SCA (bradyarrhythmias or tachyarrhythmias), and the efficacy of early defibrillation given by the WCD. Three patient groups were followed; patients listed for heart transplantation, patients with dilated cardiomyopathy (DCM) and patients using inotropes known to cause ventricular tachyarrhythmias.

## Methods

### Study design

#### Study objectives

The WIF study was designed to collect SCA events, WCD defibrillation efficacy, and WCD usage data in HF patients. The WCD usage criteria was defined as met if median daily use was at least 85% of the day in those patients that wore the device for at least 7 days. WCD prescription length was not prespecified, but was anticipated to be 3 months. The WCD (LifeVest®, ZOLL, Pittsburgh, PA) is FDA approved for use in this population, and is reimbursed by Medicare and other insurers. The study was sponsored by the manufacturer (ZOLL, Pittsburgh, PA).

#### Sites

Ten centers with WCD prescribing physicians were selected for the study based upon expressed interest. All sites were in the US and included both academic and community centers.

#### Study population

Patients were enrolled from July 1, 2007 through February 1, 2010. For the purposes of this registry, HF patients were screened if they were: 1) listed (or being considered) for heart transplantation, and/or, 2) they had DCM (with VT or EF ≤ 40%), and/or, 3) the patient was receiving inotropes. HF patients were excluded from the study if they had an active ICD or if they were impaired such that they could not use the device. Age, pregnancy, and time since HF diagnosis were not exclusion criteria. The device’s default VT and VF detection rates were set at 150 and 200 beats per minute, respectively, and the default shock energy was set at 150 joules. However, physicians had the ability to change these settings. A sample size of 500 was originally specified in order to capture multiple SCA events based on an estimated event rate of one or two percent over the three month study period. However, due to lower than expected site participation the registry was terminated after three years.

### Data collection

The protocol, informed consent document, and relevant supporting information was submitted and approved for each study site by their local institutional review board, for Saint Luke’s Hospital of Kansas City, Albert Einstein Health Care Network, and The University of Iowa, or a national institutional review board (Schulman Associates) before the study was initiated in accordance with ethical principles that have their origin in the Declaration of Helsinki and consistent with Good Clinical Practice and applicable regulatory requirements. Patient demographics, limited medical history, medications, laboratory results, and cardiac testing were collected upon entry. WCD device records were interrogated to determine compliance with use, defibrillation events, and arrhythmia detection. WCD ECG recordings, whether patient initiated or automatic, were also collected. Reasons for WCD device removal, cardiac events during the study, medications, and cardiac testing data were collected at the end of WCD use. Patient survival during WCD use was determined three months after the patient began participation. The social security death index (SSDI) was used to determine all cause-mortality after WCD use. The duration of follow-up was computed from the time of WCD start until death for those who died, and to the final date of data collection (June 9, 2010) for those who were still alive.

### Statistical analysis

Descriptive statistics were utilized to describe this population based on data collected at the time of referral for WCD therapy or after therapy. All values are reported as means ± standard deviations. Student’s *t* test (unpaired) for continuous data, or Fisher’s exact test for categorical data, was used where a *p* value of 0.05 was considered statistically significant. The Pearson’s product–moment correlation coefficient was calculated using the free online academic software at http://www.wessa.net to test whether a correlation existed between factors impacting WCD use.

## Results

The WIF registry began enrolling patients in mid July 2007. At the conclusion of the study in May 2010, 89 patients had been enrolled from ten centers. This report is based on the analysis of data from 82 patients who completed the study. Of the seven who did not complete the study, 4 were lost to study follow-up after completing WCD use, and 3 dropped out after wearing the WCD for a couple of hours. The cohort was predominantly male (72%) with a mean age of 56.8 ± 13.2 years (range: 25–82). Most patients were diagnosed with ischemic or non-ischemic dilated cardiomyopathy (DCM) with a low ejection fraction (EF) (98.8%), and twelve were listed for cardiac transplantation. Four patients were on inotropes, and three of these patients were also listed for transplantation. Baseline characteristics are shown in Table 1. The majority of patients had NYHA class II- III HF, and only one patient started out with an EF >45% (transplant rejection with VT). The etiology of the cardiomyopathy was idiopathic in 39% of patients, ischemic in 36% of patients, and a variety of other non-ischemic causes (Table 2). Co-morbidities included hypertension (68%), diabetes (44%), smoking (56%), and obesity (42%). Seventy-eight patients (95.1%) were on beta-blockers, 63 (76.8%) on ACE inhibitors, 16 (19.5%) on angiotensin II receptor blockers, and seven (8.5%) on amiodarone. Only 5 (6.1%) patients had a prior history of sustained ventricular dysrhythmias. Time since first HF diagnosis was not collected.

**Table 1:** Baseline characteristics

**Baseline characteristics**	**Total (n=82)**
Sex: Males (%)	59 (72%)
Age: Mean (SD)	61.0 (11.1)
Range	37-83
Baseline EF (SD)	23.9% (9.4%)
Range	7.5%-65%
Baseline NYHA class (%)	n=74
Class I	11 (14.9%)
Class II	20 (27.0%)
Class III	40 (54.1%)
Class IV	3 (4.1%)
Prior MI (n=78)	17 (21.8%)
Prior PCI (n=80)	10 (12.5%)
Prior CABG (n=81)	17 (21.0%)
Prior sustained VT (n=82)	4 (4.9%)
Prior VF (n=79)	1 (1.3%)
HTN (n=81)	55 (67.9%)
Diabetes (n=81)	36 (44.4%)
Smoking (n=81)	45 (55.6%)
BMI	n=69
<18.5	2 (2.9%)
18.5-24.9	20 (29.0%)
25–29.9	18 (26.1%)
>30	29 (42.0%)
ECG	n=81
Atrial Fibrillation	8 (9.9%)
Bradycardia	3 (3.7%)
Sinus rhythm with 1^st^ deg AV block	2 (2.5%)
Left bundle branch block	3 (3.7%)
Right bundle branch block	2 (2.5%)
Sinus tachycardia	12 (14.8%)
Multiple PVCs	4 (4.9%)
Device History	
Active pacemaker	2 (2.4%)
Past/inactive pacemaker	2 (2.4%)
Prior/inactive ICD	4 (4.9%)
Medications	
Beta Blockers	78 (95.1%)
ACE Inhibitors	63 (76.8%)
Angiotensin II Receptor Blockers	16 (19.5%)
Anti-arrhythmics	7 (8.5%)

**Table 2:** Primary causes of cardiomyopathy

	
Etiology	n=80
Idiopathic	31 (38.8%)
Ischemic	29 (36.3%)
Alcohol/recreational drugs	6 (7.5%)
Other NICM	5 (6.3%)
Familial	2 (2.5%)
Viral	2 (2.5%)
Hypertrophic	2 (2.5%)
Allograft Rejection	1 (1.3%)
Pharmacological	1 (1.3%)
Peripartum	1 (1.3%)

### Compliance with the WCD

The average period of WCD usage for all patients was 79.5±57.8 days (median: 79, range: 1–277). Two patients were still wearing the device at the end of the study. Compliance with wearing the WCD was then calculated based on patients who wore the device for 7 days or greater (n=75). The average daily device use was 19.5±4.6 hr/day (median: 21.8; range: 3.7-23.7) over an average of 75.1±57.7 days (median: 64; range: 7–277). Average daily use was significantly correlated to total days worn (r=0.300, p<0.01).

### Outcomes

There were no SCA events or deaths during the study, and 90- day survival after WCD fitting was 100%. There were also no adverse events or inappropriate shocks by the WCD. Two patients (2.7%) complained of palpitations, and five patients (6.8%) complained of lightheadedness or fainting during WCD use, with no sustained ventricular arrhythmias or asystoles detected by the WCD. Three patients had a CABG procedure during WCD use but none had percutaneous coronary intervention. Medication use at the end of the study was similar to baseline, with 95.7% on beta-blockers, 72.5% on ACE inhibitors, 20.3% on angiotensin II receptor blockers, and 15.9% on amiodarone. Four patients underwent an electrophysiology (EP) study after discharge. One patient had an inconclusive EP result, one had a positive result, and two had a negative test. The first two patients then underwent ICD implantation.

**Figure 1:**
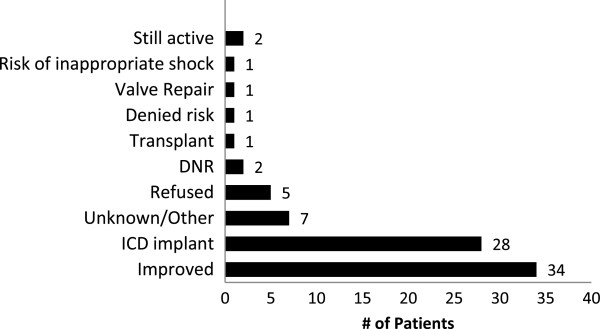
**WCD end of use reasons.** The reported reasons for ending WCD use in all patients.

At the time of WCD discontinuation, 34 (41.5%) patients were considered much improved and no longer needed the WCD due to either an improved EF (defined as EF ≥ 35% at the time use ended) (31 out of 34), acute allograft rejection resolved (1 of 34), feeling better (1 of 34), and one unknown reason. Twenty-eight (34.1%) went on to receive an ICD implant. Six (7.3%) refused to wear the device due to discomfort or other reasons, seven (8.5%) patients had an unknown/other reason for removing their WCD. Two (2.4%) transitioned to “do not resuscitate” status, one (1.2%) received a heart transplant, one (1.2%) had aortic/mitral/tricuspid valve repair, one (1.2%) had frequent inappropriate detections, and two (2.4%) patients were still using the WCD at the close of the study (Figure 1). Although a greater proportion of presumed non-ischemic patients ended WCD use due to improvement as compared to ischemic patients (50% vs. 34%), it was not statistically different (p=0.267). Likewise, 32% of non-ischemic patients received ICD after WCD use vs. 45% of ischemic patients.

#### Improved LVEF during WCD use

**Figure 2:**
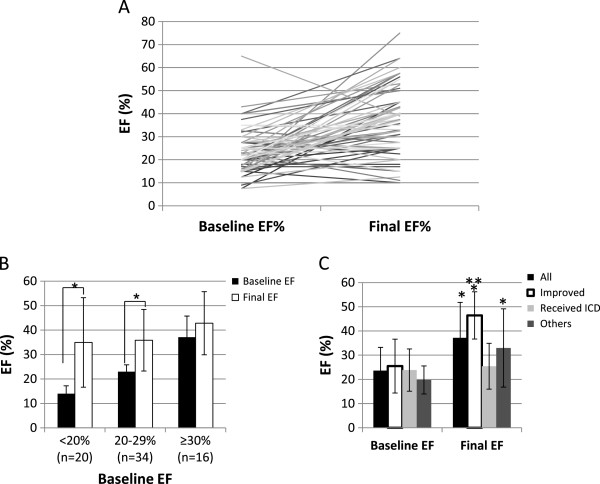
**LVEF per patient over time.** (**A**) LVEF (%) per patient at WCD initiation to WCD end. Data is shown for 70 patients who had both a baseline and final EF measurement. (**B**) LVEF improvement grouped by starting LVEF (n=70). * denotes significance of p<0.001 as compared to respective baseline EF. (**C**) Outcomes as related to initial and final LVEF. Average baseline and final LVEF (± SD) measurements are shown for all patients having both a baseline and final EF recorded (n=70), patients who improved during WCD use (n=33), patients who received an ICD after WCD use (n=20), and all remaining patients who ended use for other reasons (n=17). All groups, except those receiving ICDs, significantly improved EF during the course of WCD use. Those patients who improved had the highest overall final EF. * denotes significance of p<0.005 as compared to respective baseline EF. ** denotes significance of p<0.005 as compared to other final EF groups.

The average final EF for all patients was 37.2%±14.6% (n=70), with an average EF increase per patient of 13.5%±15.7%. This was a statistically significant improvement of EF since initial enrollment (*p*<0.001). Fifteen patients had an EF >50% at the end of WCD use. Baseline EF versus final EF per patient is shown in Figure 2(a,b). Those patients with lower baseline EF experienced the greatest improvement in EF (Figure 2b). Only one patient with a high baseline EF was enrolled by virtue of transplant rejection with VT. The average length of prescription to ICD implant was 77±55 days (median:76), while the average length of time between prescription to EF improvement was 82±58 days (median:85). Figure 2c shows change in EF according to outcome (improved, received ICD, or other). The average final EF before ICD implant was 25.5%±9.5% (n=20). The final EF of those patients who improved was 46.4%±9.8% compared to a baseline of 25.5% ± 11.1% (n=33, *p*<0.0001). Those experiencing EF improvement had an average 20.9% (or 1.8 fold) increase in their EF% from baseline. There was no difference in baseline EF between those who improved and those who did not. New York Heart Association class also improved by 0.5±1.0 points since initial enrollment (n=59, p<0.005). There were no significant differences in beta-blocker, ACE-Inhibitor, or angiotensin-II receptor blocker usage between those patients who improved, and those who received an ICD (data not shown). After WCD use, six patients (7.3%) died from unknown causes 26, 56, 229, 254, 304, and 553 days (average 288±176 days) after WCD discontinuation, according to the social security death index. The patient who died 26 days after WCD use received an ICD (EF=27.5%). The patients who died at 56 and 229 days post- WCD use had an EF improvement (EF= 43% and 50%, respectively at the time of WCD end of use). The patient who died 304 days after WCD use had stopped the WCD due to discomfort. The patient who died 553 days after WCD ended use was due to a lack of improvement (EF=31%) and switch to a “do not resuscitate” status.

#### Patients on transplant list and inotropes

Twelve patients on the heart transplant list completed the study. Four patients were able to remove the WCD after their condition improved, four received ICDs, one had aortic/mitral/tricuspid valve repair, one received a heart transplant, and two ended WCD use for an unknown reasons. Ninety day survival was 100%, although one patient died 56 days after WCD discontinuation for improved EF, according to the social security death index. Of the four patients on inotropes; two received ICDs, one received a heart transplant, and one had an improvement in EF but died 56 days after WCD discontinuation (same patient as above).

## Discussion

The intent of the WIF registry was to observe the use of the WCD in a population of HF patients over a 90 day period. The enrolled group represented a mix of dilated cardiomyopathy patients (Tables 1 and 2), with the majority of patients having an idiopathic (39%) or ischemic (36%) etiology of HF. Twelve patients (14.6%) were listed for cardiac transplantation, and four (4.9%) were on inotropes. As one may expect from the small sample size that resulted from lower than anticipated enrollment, there were no SCA events in the 82 patients who completed the study. Current WCD event statistics suggest an event rate for new cardiomyopathy patients wearing the WCD to be approximately 0.00013 VT/VF events per patient per day [18]. Therefore, the chance of not having an event was approximately 42%. Regardless, the WCD bridged the DCM patients until long-term therapy decisions could be made. There were also no adverse events or unnecessary shocks by the WCD during the study. Daily compliance with the WCD was a median of 21.8 hrs, which met our goal of at least 85% median daily wear time. This compliance is very similar to that of the current general commercial population (median=21.7 hr/day) [18]. Seventy percent of patients wore the device for average ≥ 80% of the day. Although assessing and comparing compliance is difficult, compliance with the WCD is similar to compliance with medications in HF populations [19].

Interestingly, 34 (41.5%) patients were considered much improved at the end of the study and no longer needed the WCD, mostly due to an improved EF (EF ≥ 35%). This may have been due to the high rate of beta-blocker use, or possibly because some patients had reversible causes of disease. However, 33% of the 12 transplant-listed patients also showed improvement. To our knowledge, few recent peer-reviewed studies have analyzed improvement in ventricular function 60–90 days post-diagnosis or acute decompensation of DCM [20,21]. The IMAC trial, which studied intravenous immune globulin in recent onset DCM, also saw mean LVEF increase significantly in patients from 25% to 41% over a 6 month period regardless of treatment [22]. It should also be emphasized that improvement in LV function should not be taken as a “cure” and medications should be continued or monitored closely, as relapses may occur [21]. Although we do not know the cause of death, two patients who were discontinued from the WCD due to EF improvement (EF=43% and 50%) died (56 and 229 days) after removing the WCD. There was an overall 7.3% mortality rate over 288±176 days after being fit with the WCD. Unfortunately, this time period was beyond the scope of the study and the reasons for death are unknown. The mortality rate is similar to that seen in the SCD-HeFT and DEFINITE (non-ischemic) trials [7,9].

The benefits of prophylactic ICD implantation remains contentious in DCM patients [14]. Although ICDs have been shown to be beneficial in this group of patients [7,8], it is not without risk, and the event rate remains low (especially for those without prior MI) [23]. For this reason, risk stratification of these patients remains extremely important. A recent study showed a yearly increase in out-of-hospital mortality rates in Medicare HF patients, possibly due to shortened hospital stays [24]. It may be beneficial to the patient (and society) to give the patient enough time to potentially stabilize on medication and recover LV function, while still keeping them protected and out of the hospital. It may also be beneficial to avoid ICD placement in the transplant-listed patient, if their wait is not expected to be extremely long. These goals can be accomplished with the WCD.

### Study limitations

There were several limitations to this study. First, due to the smaller than expected enrollment, the study did not meet its primary objective of collecting SCA incidence data. Also, 8.5% of those who were initially enrolled were lost to study follow-up. Second, factors affecting EF improvement could not be analyzed fully due to the heterogeneic etiologies and unknown duration of disease. Finally, the mortality rate in our study was determined using the Social Security Death Index and may not have captured all deaths in our population, leading to erroneous conclusions based on this data

## Conclusions

In conclusion, the WCD monitored HF patients until further assessment of risk. The leading reasons for end of WCD use were improvement in left ventricular ejection fraction (LVEF) or ICD implantation if there was no significant improvement in LVSF. Due to the smaller than anticipated sample size, there were no SCD events in the 82 patients who completed the study (≤90 days). The overall 7.3% mortality rate over 288±176 days after being fit with the WCD is similar to that seen in other trials.

## Competing interests

NB and SS are employees of ZOLL. ZOLL sponsored this study, including the article-processing charge of this manuscript, and contributed to study design, implementation, and data monitoring.

## Authors’ contributions

AK and SS contributed to study design. All authors contributed to the acquisition of data and analysis of the results. AK, DK, NB, and SS were involved in drafting the manuscript. All authors revised the manuscript critically and approved the final version.

## Disclosures of financial support

This research was sponsored by ZOLL Cardiac Management Solutions, Pittsburgh, PA.

## Pre-publication history

The pre-publication history for this paper can be accessed here:

http://www.biomedcentral.com/1471-2261/12/123/prepub
